# Skeletal Maturity in Adolescence: Evaluating Bone Development and Age Metrics

**DOI:** 10.3390/diagnostics15080970

**Published:** 2025-04-10

**Authors:** João Pinheiro, Luís Ribeiro, Diana Teixeira, Anabela Ribeiro, Manuel João Coelho-e-Silva

**Affiliations:** 1 School of Health, Gambelas Campus, University of Algarve, 8005-139 Faro, Portugal; jppinheiro@ualg.pt (J.P.); amribeiro@ualg.pt (A.R.); 2 Centre for Health Studies and Development, School of Health, Gambelas Campus, University of Algarve, 8005-139 Faro, Portugal; 3 Sport Physical Activity and Health Research & Innovation Center (SPRINT), 2001-904 Santarém, Portugal; 4 Research Unit for Sport and Physical Activity, Faculty of Sport Sciences and Physical Education, University of Coimbra, 3004-531 Coimbra, Portugal; mjcesilva@hotmail.com; 5 NOVA Medical School, Medical Sciences Campus, University Nova of Lisboa, 2829-516 Caparica, Portugal; diana.teixeira@nms.unl.pt

**Keywords:** bone age, chronological age, bone mineral content, bone mineral density, body composition

## Abstract

**Background/Objectives**: Bone maturation and development are crucial for growth and development, especially in children and adolescents; however, some qualitative methods, such as Greulich & Pyle, do not provide accurate data. Our aim is to verify whether skeletal age (SA) can predict and correlate with bone mineral content (BMC), bone mineral density (BMD), and body composition (BC). **Methods**: A cross-sectional study was conducted on 115 male adolescents (ages 12.1–15.8 years). Skeletal age was assessed using the Tanner–Whitehouse 3 (TW3) method, while BMC, BMD, and BC were measured using full-body DXA. Anthropometric data, including height and body mass, were also recorded. Statistical analysis included descriptive methods and bivariate correlation coefficients. **Results**: SA was significantly correlated with stature (r = 0.598, *p* = 0.001) and body mass (r = 0.517, *p* = 0.001), showing a stronger association than chronological age (CA) for these variables. Body composition variables, including lean mass (LM) (r = 0.521, *p* = 0.001) and fat tissue (FT) (r = 0.522, *p* = 0.001), also showed a stronger correlation with SA than CA. However, associations between SA and bone parameters were weaker: BMC (r = 0.103, *p* = 0.275) and BMD (r = 0.161, *p* = 0.086) did not reach statistical significance. When stratified by SA/CA tertiles, individuals in the highest tertile exhibited slightly greater BMC (1439 ± 108.32 g) and BMD (1.028 ± 0.127 g/cm^2^), though without a significant effect. These findings suggest a dynamic but complex relationship between skeletal age and bone development. **Conclusions**: SA demonstrates a stronger association with anthropometric and body composition variables than CA, highlighting its potential as a predictor of growth used in conjunction with LM and FM. However, its relationship with BMD and BMC remains inconclusive, warranting further longitudinal research, considering limitations regarding nutritional intake.

## 1. Introduction

Understanding the growth and development of children and adolescents requires a detailed examination of the biological principles that govern human maturation [1,2]. Biological maturation is a highly individualized process that affects all tissues, organs, and systems of the human body [3,4] and is influenced by a variety of interdependent factors, including genetic/epigenetic mechanisms, psychosocial and environmental interactions, stress, and caloric intake [4,5]. This process is primarily regulated by the hypothalamic-pituitary-gonadal axis, which drives endocrine and skeletal maturation [6]. However, despite the well-established role of biological maturation in physical development [7], the precise relationship between maturational timing (early, on-time, or late) and participation in physical activity (PA) remains a subject of debate [2,8].

Biological Maturation

The assessment of biological maturation relies on multiple indicators, each with distinct advantages and limitations. Among the most widely used methods are peak growth velocity age, pubertal development scales, percentage of predicted adult height, Tanner stages, estradiol level analysis, and skeletal age (SA) determination [2,4,8,9,10]. SA, which reflects the degree of ossification and bone maturity, is one of the most reliable indicators of biological maturation. It is commonly used to evaluate growth abnormalities, delayed or early puberty, and deviations in physical development [4,5,6,7,8]. Since chronological age (CA) alone is insufficient to determine an individual’s developmental stage, SA assessments provide a more precise evaluation of growth trajectories from childhood through adolescence [4]. Unlike other methods that are restricted to puberty, SA offers a continuous measure of maturational progress, allowing for long-term monitoring of skeletal development [4].

Skeletal Age

The determination of SA is based on radiographic evaluation of the hand and wrist, following a predictable sequence of bone maturation [5]. The Greulich & Pyle (GP), Tanner–Whitehouse (TW3), and Fels methods are the most widely used techniques for estimating skeletal age [5,6,7,8]. Each method is based on reference populations with different socioeconomic and ethnic backgrounds, affecting their applicability across diverse groups. The GP method involves comparing radiographs to standard reference images but does not account for variations in the maturity of individual bones [11]. In contrast, the TW3 method, used in this study, assigns SA based on the maturation of specific bone groups, including the radius, ulna, metacarpals, and phalanges [5,11]. Despite the clinical utility of SA assessment, its interpretation requires specialized training, and variations between assessment protocols can impact accuracy. Additionally, concerns regarding radiation exposure have led to increasing interest in alternative imaging techniques; however, the radiation dose in standard hand-wrist radiographs is minimal (0.001 mGy), far below background radiation levels [4,5].

Clinical Applications

Beyond clinical applications, SA has been proposed as a useful tool in sports science, particularly for talent selection, injury risk assessment, and performance monitoring [4,12,13,14,15,16]. The association between SA and body composition variables such as lean mass (LM) and fat mass (FM) has gained attention, as these factors contribute to bone mineral content (BMC) and bone mineral density (BMD) [17,18]. During growth, skeletal modeling occurs through a dynamic process of bone deposition and resorption, with peak bone mass (PBM) estimated to be 90% complete by the end of adolescence [15,16]. While pubertal timing plays a crucial role in determining PBM, the relationship between SA and bone parameters remains unclear, with some evidence suggesting that early maturation is linked to higher BMD values [17,18]. However, it remains uncertain whether maturational differences lead to long-term advantages in bone strength [19,20,21,22,23].

Objective and Research Hypothesis

Although previous studies have explored the link between biological maturation and body composition, few have specifically examined the correlation between SA, BMD, and BMC in adolescent males [14,15,24,25,26,27,28]. This study aims to analyze SA as a predictor of bone mineralization and body composition, focusing on lean mass and fat mass, using DXA scans for precise measurement. By investigating these relationships, we seek to clarify the role of skeletal maturation in bone health and growth, contributing to a better understanding of developmental processes in adolescence.

## 2. Materials and Methods

The participants of this cross-sectional observational study were recruited voluntarily in school groups in the Algarve region of Portugal. The study was conducted in accordance with the recommendations of the Helsinki Declaration for Human Studies, having been previously approved by the Ethics Committee of the Faculty of Sport Sciences and Physical Education of the University of Coimbra (CE/FCDEF-UC/00172016). All participants were informed about the objectives, experimental protocol, and procedures of the study and informed that participation was voluntary and that they could abandon the study at any time. Informed consent was obtained from the parents or legal guardians. The imaging evaluations were carried out in an accredited imaging laboratory by qualified radiographers.

Sample

The sample consisted of 115 male Caucasian subjects, with chronological ages between 12 and 16 years to encompass peak growth stages. The following inclusion criteria were considered: (i) male; (ii) chronological age of less than 16 years; (iii) without any federated sports practice at the date of the start of data collection or in the last three years. Regarding the exclusion criteria, the following were considered: (i) previous fractures of long bones or any surgery that leads to immobilization for a period of more than 6 months; (ii) metabolic pathologies that affect bone tissue and body composition, such as diabetes; (iii) current or previous medical condition known to affect growth, maturation, physical activity, or nutritional status, and medications known to affect growth, maturation, or bone mineral accrual such as steroids; (iv) individuals with indwelling hardware; abnormalities of the skeleton or spine such as scoliosis 20 degrees or more, kyphosis, or skeletal dysplasia by history; and (v) participation in a diet or exercise intervention study in the previous year were also excluded from participation.

Bone Age

Bone age was determined by radiological examination of the left hand and wrist. To ensure accurate data interpretation, it is crucial to follow the proper procedure. The hand, typically the left one (as it is often less affected by daily activities in right-handed individuals), should be positioned palm-side down on a rigid surface with the fingers spread apart for an anteroposterior radiograph. The middle finger should align with the axis of the forearm, and the X-ray tube’s center should be directed at the distal end of the third metacarpal. A recommended distance of 76 cm between the X-ray tube and the hand should be maintained. The fingers should be spaced apart to avoid overlap, and the thumb should naturally rotate approximately 30° away from the first finger. Additionally, the X-rays must be perpendicular to the rigid plane for optimal imaging. Bone age was determined using the Tanner–Whitehouse 3 (TW3) method [29,30,31]. The equipment used was the Siemens Axiom Aristos VX—model no. 1350S of conventional radiology with 45 kVp, 6 mA (Siemens Healthcare, Erlangen, Germany). The approximate effective radiation dose for the subjects was around 0.001 mGy.

Anthropometry

A single experienced observer performed anthropometric procedures according to standardized procedures [32,33,34,35]. In summary, the individuals were weighed with as little clothing as possible and without shoes, and height was measured at approximately 0.1 cm using a scale (Tanita BC610, Amsterdam, The Netherlands) and a stadiometer (Seca B213, Hamburg, Germany). Chronological age was calculated as the difference between date of birth and measurement.

Dual-energy X-ray absorptiometry

A single dual-energy x-ray absorptiometer (DXA) (GE Lunar Prodigy, software v.11.03) was used, and the regions of interest were manually positioned according to the manufacturer guidelines and the International Society for Clinical Densitometry [34]. The long-term calibration stability was monitored every day when exams were performed. The precision error for BMD and BMC was less than 1% for the spine phantom and less than 2% for the whole-body phantom.

The procedure allows for the measurement of segmental composition: arm, leg, and trunk. The primary outcomes were bone mineral content (BMC), bone mineral density (BMD), fat tissue (FT), and lean mass (LM) for the whole body (WB) and specific regions.

Data Analysis

All statistical analyses were conducted using SPSS 26.0 (IBM, Chicago, IL, USA) and GraphPad Prism V.10.0 for Windows (GraphPad Software, Boston, MA USA, www.graphpad.com). Descriptive and inferential statistical methods were applied to examine the relationships between SA, BMC, BMD, and BC variables.

Descriptive statistics provided measures of central tendency (mean), dispersion (standard deviation), and range, to summarize key variables, CA, SA, height, BM, FT, LM, BMC, and BMD.

Bivariate correlation analysis was performed using Pearson correlation coefficients (r) to assess the relationships between SA, CA, and BC measures. The SA/CA ratio was also analyzed as an independent variable to examine its influence on skeletal and body composition indicators.

Generalized linear models were applied to determine whether SA and SA/CA tertiles had significant effects on BMC, BMD, and other body composition variables and pairwise comparisons and effect sizes (Cohen’s d) were used to evaluate differences between maturational groups for a confidence interval (CI) of 95%. For significance thresholds, a significance level of 5% (*p* < 0.05) was used for most analyses, with a more conservative threshold of 1% (*p* < 0.01) applied where multiple comparisons were performed.

Limitations

One of the key limitations of this study is the lack of nutritional intake assessment, which is a critical factor influencing BMC, BMD, and overall BC. Adequate intake of calcium, vitamin D, protein, and other micronutrients is essential for optimal bone mineralization, particularly during adolescence, a period of rapid skeletal growth. The study did not account for known determinants of bone accretion, such as dietary intake and physical activity levels, which can significantly influence BMC and BMD. However, since this limitation applied to all participants, its impact on the overall findings may be minimal.

No hormonal measurements (e.g., testosterone, estrogen, growth hormone) were included, which could have provided additional insights into the biological mechanisms influencing bone maturation and body composition.

Since this is a cross-sectional study, it only provides a snapshot of the relationship between SA, bone health, and BC. A longitudinal approach would be necessary to track changes over time and establish causality.

The sample consisted exclusively of male Caucasian adolescents from a specific geographic region (Algarve, Portugal), limiting the generalizability of the findings to other populations, including females and individuals from diverse ethnic or socioeconomic backgrounds.

## 3. Results

Anthropometric Characteristics, Body Composition, and Bone Mineral Parameters

The study included 115 male adolescents between the ages of 12.1 and 15.8 years, with SA ranging from 11.9 to 16.5 years (Table 1). The mean CA was 14.05 ± 0.93 years, whereas SA was slightly higher at 14.27 ± 1.08 years, indicating variability in biological maturation within the sample. Stature varied between 126.6 cm and 183.7 cm, with a mean stature of 162.13 ± 11.02 cm, and BM ranged from 31.0 kg to 82.7 kg, with an average of 53.0 ± 11.19 kg. FT showed considerable variation, ranging from 11.9 cm to 31.6 cm, with a mean of 20.2 ± 4.28 cm, and LM (indicative of muscle mass) varied between 22.7 kg and 60.7 kg, with a mean value of 38.9 ± 8.22 kg. BMC ranged from 1254 g to 1689 g, with an average of 1439 ± 108.32 g, and BMD values were between 0.723 g/cm^2^ and 1.362 g/cm^2^, with a mean of 1.028 ± 0.127 g/cm^2^.

Pearson and Simple Bivariate Correlation Coefficients

Table 2 presents the Pearson correlation coefficients (r) between SA, CA, and key morphological and BC variables, including stature, BM, FT, LM, BMC, and BMD. These correlations provide insights into how skeletal maturation is associated with body size and bone health.

Stronger Correlations Between SA and Morphological Variables

We found stronger correlations between SA and morphological variables; stature showed the highest correlation with SA (r = 0.598, *p* = 0.001), indicating that SA is a strong predictor of stature. SA also had a significant positive correlation with BM (r = 0.517, *p* = 0.000), suggesting that more biologically mature individuals tend to have a higher body weight. Comparatively, CA showed weaker correlations with stature (r = 0.397, *p* = 0.000) and BM (r = 0.484, *p* = 0.000), reinforcing that SA is a better indicator of physical development than CA alone.

Body Composition Correlations

For BC correlations, FT and LM both had a stronger correlation with SA (r = 0.522, *p* = 0.000) than with CA (r = 0.491, *p* = 0.001), suggesting that biological maturation is more closely linked to body composition changes, particularly in lean mass accumulation.

Weak and Non-Significant Correlations Between SA and Bone Parameters (BMC & BMD)

The correlation between SA and BMC was low (r = 0.103, *p* = 0.275) and non-significant, indicating that bone mineral content does not strongly correlate with skeletal maturation in this sample. Similarly, SA had a weak correlation with BMD (r = 0.161, *p* = 0.086), failing to reach statistical significance. CA showed equally weak relationships with BMC (r = 0.041, *p* = 0.661) and BMD (r = 0.150, *p* = 0.109), suggesting that neither CA nor SA alone is a strong predictor of bone mineralization.

SA/CA Ratio as an Indicator of Maturational Cadence

The SA/CA ratio correlated strongly with stature (r = 0.562, *p* = 0.000), reinforcing its potential use as a biological maturation indicator. However, it showed only small or trivial correlations with body composition variables and bone parameters, further supporting the idea that additional factors, such as nutrition and physical activity, play a role in bone development.

Summary of key findings

- SA is a better predictor of stature, body mass, and body composition than CA;
- MC and BMD do not show significant correlations with SA or CA, suggesting that bone mineralization may depend on other factors beyond skeletal maturation;
- The SA/CA ratio effectively captures maturational differences, but its relationship with bone health remains inconclusive.

SA/CA tertiles for Anthropometric, BC, and Bone Variables

The use of SA/CA tertiles in this study serves as a valuable tool for better understanding the relationship between skeletal maturation and key physical and biological characteristics, such as stature, BM, BC, and bone parameters. By grouping participants based on their SA/CA ratio, rather than treating SA and CA as separate continuous variables, this method allows for a more nuanced analysis of maturational differences.

Effect of SA/CA Tertiles on Stature and Body Mass

**Figure 1:**
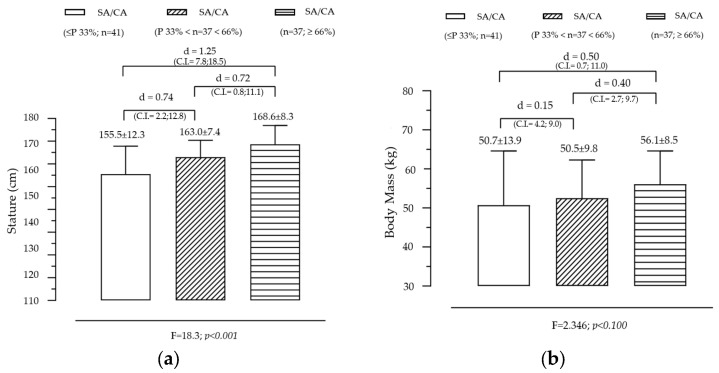
Variation in body size according to the maturational state given by SA/CA in male adolescents for stature (**a**) (F = 18.3; *p* = 0.001) and body mass (**b**) (F = 2.35; *p* = 0.100), (*n* = 115).

Figure 1 illustrates the variation in stature and body mass across groups created by SA/CA tertiles, highlighting the significant effect of maturational status on stature and a non-significant but observable trend for BM. The analysis revealed a significant effect of SA/CA tertiles on stature (F = 18.253, *p* = 0.000), indicating that individuals in higher maturational groups tend to be taller. The tertile-based grouping of SA/CA revealed a clear progression (G1 < G2 < G3) in stature, with the mean height increasing as individuals were categorized into higher SA/CA groups. Pairwise comparisons showed that G1 (lowest SA/CA tertile) was significantly shorter than G2 (moderate maturational status), with a moderate effect size (d = 0.74). G1 was also significantly shorter than G3 (highest SA/CA tertile), with a large effect size (d = 1.25). G2 was shorter than G3, with a moderate effect size (d = 0.72). These results suggest that skeletally advanced individuals (higher SA/CA ratio) are significantly taller, reinforcing the idea that biological age is a better predictor of growth than chronological age alone. Although the overall effect of SA/CA tertiles on body mass was not statistically significant (F = 2.35, *p* = 0.100), a clear increasing trend was observed. Individuals in G3 (highest SA/CA tertile) had a mean body mass of 56.1 kg, compared to 50.7 kg in G1 (lowest tertile), representing a difference of 5.4 kg. The effect size (Cohen’s d = 0.50) between G1 and G3 was small, indicating that while more biologically mature individuals tend to have higher BM, the variability within groups may have reduced statistical significance.

Summary of key findings

- Stature is strongly influenced by skeletal maturation, confirming that SA is a more reliable measure for assessing growth potential than CA alone.
- The trend in body mass suggests that maturational status influences weight gain, but other factors such as nutrition, physical activity, and genetic predisposition may contribute to individual differences, explaining the lack of statistical significance.
- The large effect sizes in stature differences support the use of SA/CA ratios for evaluating maturational progress in growth studies, clinical applications, and talent identification in sports.

Effect of SA/CA Tertiles on Fat Tissue and Lean Tissue

**Figure 2:**
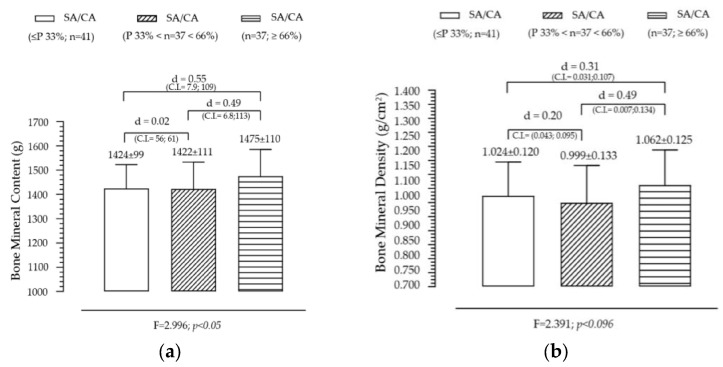
Variation of fat tissue (**a**) and lean soft tissue (**b**) according to the maturational state given by SA/CA in male adolescents (*n* = 115).

Figure 2 presents the variation in fat tissue and lean soft tissue across the groups created by the SA/CA tertiles, aiming to assess whether skeletal maturation status influences body composition. FT shows a non-significant but gradual trend across SA/CA tertiles. Although no statistically significant effect was found for FT across the tertiles, a progressive increase was observed in the mean fat tissue values as SA/CA increased. This suggests that individuals in higher maturational groups (G3) tend to have slightly more fat mass than those in the lower tertiles (G1 and G2), but the differences are not substantial enough to reach statistical significance. LT shows a positive but modest relationship with maturational status, exhibiting an increasing trend across tertiles, with individuals in G3 (highest SA/CA ratio) having more lean mass than those in G1 (lowest SA/CA ratio). However, like FT, this effect was not statistically significant, indicating that maturation alone does not fully explain variations in lean mass accumulation.

Summary of key findings

- The lack of statistical significance suggests that skeletal maturation alone is not the primary driver of body composition changes.
- The slight increase in fat tissue across maturational groups might be linked to natural pubertal changes in body fat distribution, particularly in adolescents undergoing later stages of maturation.
- The incremental increase in lean soft tissue is consistent with the role of testosterone and growth hormone in muscle development, though this study did not include hormonal data to further analyze this effect.

Effect of SA/CA Tertiles on Fat Tissue and Lean Tissue

**Figure 3:**
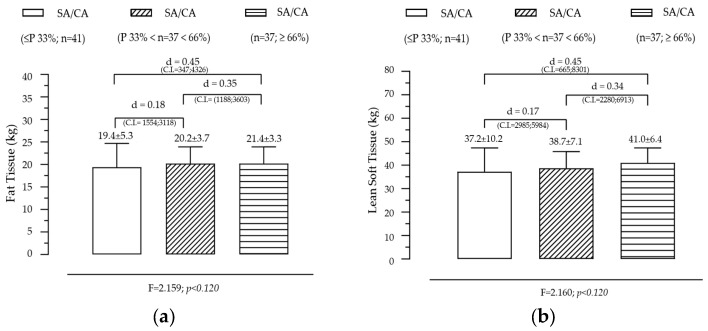
Variation of BMC (**a**) and BMD (**b**) according to the maturational state given by SA/CA in male adolescents (*n* = 115).

Figure 3 illustrates the variation in BMC and BMD across SA/CA tertiles, aiming to assess whether skeletal maturation status significantly influences bone mineralization. BMC shows a non-significant but gradual increase across SA/CA tertiles. The mean BMC values increased progressively across the tertiles (G1 < G2 < G3), with individuals in the highest SA/CA tertile (G3) having a slightly higher BMC than those in the lower tertiles (G1 and G2). However, this difference was not statistically significant (F = 2.996, *p* = 0.05), suggesting that while maturational progression might influence BMC, other factors such as nutritional intake, physical activity, and genetic predisposition likely contribute to bone mass accumulation. Pairwise comparisons showed small effect sizes between groups, with G1 vs. G3 (d = 0.50) and G2 vs. G3 (d = 0.49), indicating a trend but without substantial statistical impact.

BMD also follows an increasing trend across SA/CA tertiles. BMD values showed a progressive increase across the SA/CA tertiles, with the highest tertile (G3) displaying a greater BMD than the lower tertiles. Although the effect of maturational status was not statistically significant, a notable difference in mean BMD values was observed between G2 (BMD = 0.999 g/cm^2^) and G3 (BMD = 1.062 g/cm^2^), with a small effect size (d = 0.49). This suggests that higher skeletal age relative to chronological age may be associated with greater bone density, though the variability within the groups might have prevented statistical significance from being reached.

Summary of key findings

- The non-significant but positive trends in BMC and BMD suggest that skeletal maturation influences bone mineralization, but additional factors contribute to individual differences.
- Lean mass and physical activity are known to play critical roles in bone accrual, which might explain why BMC and BMD did not show strong associations with SA/CA tertiles alone.
- Since BMD is a key determinant of long-term bone health, further research should explore whether higher SA/CA ratios in adolescence predict stronger bone structure in adulthood.
- These findings suggest that bone age assessments could be incorporated into pediatric evaluations to monitor bone development and identify individuals at risk of lower bone density early on.

## 4. Discussion

This study examined the relationship between SA, BMC, BMD, and BC in adolescent males. Although SA demonstrated strong correlations with stature and body mass, its associations with BMC and BMD were weak and non-significant. These findings suggest that while skeletal maturation influences overall growth, its direct impact on bone mineralization may be less pronounced or confounded by other factors such as physical activity levels, nutritional intake, and genetic predisposition.

The correlation between SA and BMC (r = 0.103, *p* = 0.275) and SA and BMD (r = 0.161, *p* = 0.086) was weak and did not reach statistical significance. This indicates that biological maturation alone is not a primary determinant of bone mineralization. Similarly, no significant effects were found for SA/CA tertiles on BMC and BMD, despite a non-significant increasing trend across maturational groups. These results align with previous studies suggesting that while SA provides a reliable indicator of growth, bone mineralization is influenced by additional factors, including weight-bearing activity and nutritional intake [15,16,36,37,38,39,40]. Furthermore, FT and LT showed non-significant variations across SA/CA tertiles, reinforcing the idea that maturational status alone does not dictate body composition changes. Previous research has highlighted that lean mass accumulation is closely tied to both maturational progress and physical activity levels, but our study did not control for PA, which may explain the lack of significant findings [24,27,41]. The classification of individuals into SA/CA tertiles provides a valuable framework for identifying those at potential risk of compromised bone health, particularly late maturers [42,43,44,45,46]. Given that SA lagging behind CA is often associated with delayed bone mineral accrual, individuals in the lowest SA/CA tertile may be at a heightened risk of lower BMD and increased susceptibility to fractures later in life [1,3,4,47]. Future research should incorporate detailed physical activity assessments to determine whether SA is an indirect predictor of body composition through its association with activity levels.

Our findings provide new insights into the relationship between SA, BMC, BMD, and BC in adolescent males. While our results suggest a non-significant association between SA and BMC/BMD, they contrast with previous research indicating that early-maturing individuals tend to exhibit greater bone mineralization. For example, studies have shown that early-maturing adolescents generally reach a higher PBM compared to late-maturing peers, which has been attributed to increased exposure to growth hormones and sex steroids, particularly estrogen and testosterone [17,18]. These hormonal influences stimulate bone formation and mineral accrual, contributing to higher BMD values in early maturers. However, unlike those studies, our research did not assess endocrine markers, which may have contributed to the lack of a significant relationship between SA and bone parameters. Future research should incorporate hormonal profiling to determine whether SA correlates with estrogen and testosterone levels, which are known to influence BMD and BMC during puberty [12,17,28]. Additionally, previous longitudinal studies have identified a strong association between SA and bone mineralization, with skeletal maturation serving as a key determinant of PBM later in life [16,42,48]. In contrast, our study utilized a cross-sectional design, which may have limited our ability to detect long-term trends in bone accrual. Since bone remodeling is a dynamic process influenced by cumulative growth patterns, future studies should track participants over several years to assess whether SA predicts BMD and BMC in adulthood [4,47]. Regarding BC, our results indicate that SA was more strongly associated with LT than FT. This contrasts with prior studies demonstrating that early-maturing individuals tend to have greater LT and reduced FT due to higher levels of androgens and increased muscle development [24,26,43,49]. Likewise, late maturers frequently present with lower muscle mass and reduced mechanical loading on bones, compounding the risk of inadequate bone strength [2,15,36,41]. One potential explanation for the weaker associations observed in our study is the lack of PA data, as previous research has shown that PA positively influences LT and BMD, particularly in adolescents engaged in weight-bearing exercise [3,6,10,37,38]. The omission of PA levels as a confounding factor may have diluted the observed relationships between skeletal maturation and BC parameters. Future studies should integrate PA assessments, such as accelerometry or structured exercise monitoring, to determine whether SA is an indirect predictor of lean mass via PA engagement [6,8,9,50]. Another important consideration is the role of nutritional intake in bone development. While our study did not include dietary assessments, previous research has demonstrated that calcium, vitamin D, and protein intake significantly influence BMC and BMD [15,16,44,45]. Adolescents with higher SA may consume greater amounts of these nutrients, supporting enhanced bone mineralization. However, the lack of nutritional data in our study limits our ability to determine whether dietary factors contributed to the observed trends. Future research should incorporate dietary recalls or biochemical markers to examine the interaction between nutritional intake, skeletal maturation, and bone health [15,16,50,51,52,53]. Finally, previous studies have also highlighted population differences in bone development, noting that genetic, ethnic, and socioeconomic factors influence the timing of skeletal maturation and BMD accrual [5,46,49]. Since our sample consisted exclusively of male Caucasian adolescents from Portugal, the generalizability of our findings is limited. Prior research has found that bone density and growth trajectories vary significantly between ethnic groups, with some populations exhibiting delayed or accelerated skeletal maturation due to genetic predisposition [46]. Expanding the sample to include diverse ethnic backgrounds would provide a more comprehensive understanding of maturational differences in bone health [5,46,49].

Recommendations for Future Research

Given the limitations of this study, future research should adopt a longitudinal design to track changes in BMD, BMC, and body composition over time, as maturational influences on bone development may become more evident in late adolescence and early adulthood [4,47]. Nutritional assessments, such as dietary recall or micronutrient analysis, should be integrated to examine the role of calcium, vitamin D, and protein intake in bone mineralization [15,16,44,45]. Physical activity measurements, such as accelerometry or structured exercise assessments, should be included to determine whether SA serves as an indirect predictor of BMC/BMD through its influence on PA levels [6,8,9,50,53]. Hormonal markers, such as testosterone, estrogen, and growth hormone, should be assessed to better understand the physiological mechanisms driving bone mineralization and body composition changes in maturing adolescents [12,17,28]. Furthermore, the sample should be expanded to include diverse ethnic and socioeconomic backgrounds, as skeletal maturation rates and bone health indicators can vary across populations due to genetic and environmental factors [5,46,49].

## 5. Conclusions

This study highlights the significant role of SA as a predictor of anthropometric variables and body composition, demonstrating stronger correlations with stature, body mass, and lean mass compared to CA. However, the relationship between SA and bone parameters such as BMC and BMD remained weak and statistically insignificant, suggesting that other biological and environmental factors contribute to bone mineralization during adolescence. Given these findings, specific recommendations include clinical and pediatric applications: SA assessment should be integrated into pediatric growth monitoring programs as it provides a more precise measure of biological development than CA alone, particularly in cases of growth disorders. In sports science and talent identification, incorporating SA assessments into youth sports selection and training programs can enhance athlete development and injury prevention. By grouping athletes based on maturational status rather than CA, training strategies can be better tailored to their physiological readiness, reducing the risk of growth-related injuries and overtraining. Additionally, SA assessments can help adjust resistance training loads, ensuring that strength and conditioning programs are aligned with an athlete’s bone and muscle development, ultimately optimizing performance outcomes and long-term athletic potential. Future research should incorporate dietary intake and physical activity data to explore how external factors influence bone health and peak bone mass development, particularly in adolescence. Further longitudinal studies are also needed to assess the long-term impact of skeletal maturation on adult bone density and fracture risk, which could inform osteoporosis prevention strategies. Clinically, SA/CA tertiles could serve as a screening tool to identify individuals in need of targeted interventions, such as nutritional optimization (calcium and vitamin D supplementation), resistance training programs, or hormone-based therapies when necessary.

At a theoretical level, these findings reinforce the biological relativity of age, emphasizing that CA alone is an inadequate metric for assessing developmental status. Instead, SA provides a more nuanced and individualized understanding of growth and maturation. Integrating SA with hormonal profiles and PA data may provide deeper insights into the maturational influences on bone health, helping to clarify how endocrine factors and mechanical loading interact to shape bone mineral accrual and density across different maturation groups. This study contributes to the growing recognition that biological maturity should be considered a key determinant in clinical, athletic, and public health frameworks, bridging gaps between pediatric endocrinology, sports medicine, and skeletal research.

## Figures and Tables

**Table 1:** Descriptive statistics for the entire sample (*n* = 115).

		Amplitude		Mean	Standard Deviation
		Minimum	Maximum		
Chronological age	Years	12.14	15.75	14.05	0.934
Skeletal age	Years	11.90	16.05	14.27	1.08
Stature	cm	126,.6	183.7	162.13	11.02
Body mass	kg	31.0	82.7	53.0	11.19
Fat tissue	cm	11.9	31.6	20.2	4.28
Soft lean tissue	kg	22.7	60.7	38.9	8.22
Bone mineral content	g	1254	1689	1439	108.32
Bone mineral density	g/cm^−2^	0.723	1.362	1.028	0.127

**Table 2:** Simple bivariate correlation coefficients between morphology given by body size and composition, including bone mineral content and density (*n* = 115).

	Chronological Age		Skeletal Age		SA/CA *	
	*r*	*p*	*r*	*p*	*r*	*p*
Stature	0.397	0.000	0.598	0.000	0.562	0.000
Body mass	0.484	0.000	0.517	0.000	0.211	0.024
Fat tissue	0.491	0.001	0.522	0.000	0.210	0.024
Lean tissue	0.491	0.001	0.521	0.000	0.210	0.024
Bone mineral content	0.041	0.661	0.103	0.275	0.157	0.099
Bone mineral density	0.150	0.109	0.161	0.086	0.076	0.421

* SA/CA (ratio of skeletal age divided by chronological age).

## Data Availability

The datasets used and/or analyzed during this study are available from the corresponding author upon reasonable request.
